# Symbiotic Bacterium-Derived Organic Acids Protect *Delia antiqua* Larvae from Entomopathogenic Fungal Infection

**DOI:** 10.1128/mSystems.00778-20

**Published:** 2020-11-17

**Authors:** Fangyuan Zhou, Letian Xu, Xiaoqing Wu, Xiaoyan Zhao, Mei Liu, Xinjian Zhang

**Affiliations:** a Shandong Provincial Key Laboratory of Applied Microbiology, Ecology Institute, Qilu University of Technology (Shandong Academy of Sciences), Ji’nan, China; b State Key Laboratory of Biocatalysis and Enzyme Engineering, School of Life Sciences, Hubei University, Wuhan, China; University of Massachusetts Medical School

**Keywords:** colonization resistance, insect-microbe symbiosis, defensive association, mutualism

## Abstract

Colonization resistance, i.e., the protective effects of associated microbiota for the animal host against pathogen infection, has been studied widely over the last 100 years. However, few molecules mediating colonization resistance have been identified. In the symbiosis formed by Delia antiqua and its associated microbes, six bacteria protect larvae from infection with the entomopathogen Beauveria bassiana, providing an ideal model to investigate the chemical mechanism for colonization resistance. Subsequently using this symbiotic system, we first compared effects of the six bacterial species, and one control bacterium (Klebsiella oxytoca) that showed no antifungal effects, on B. bassiana and its infection of *D. antiqua*. Second, metabolomic profiles of the six bacteria and K. oxytoca were compared to identify candidate metabolites that may prevent infection. Third, the concentrations of candidate metabolites *in situ* from axenic and nonaxenic larvae were determined. Finally, effects of artificial metabolite cocktails on *B.* bassiana and its infection of *D. antiqua* larvae were determined. Results showed that compared to K. oxytoca, the six bacteria produced a metabolite cocktail showing inhibitory effects on conidial germination, mycelial growth of *B.* bassiana, and fungal infection. Our work revealed novel molecules that mediate colonization resistance, which could help in developing chemical mechanisms of colonization resistance. Moreover, this work may aid in discovery and expansion of new bioactive antibiotics, promoting development of prophylactic and therapeutic approaches for treating infectious diseases.

**IMPORTANCE** The protection of associated microbiota for their animal hosts against pathogen infection has been studied widely over the last 100 years. However, how those microbes protect the animal host remains unclear. In former studies, body surface microbes of one insect, Delia antiqua, protected the insect larvae from infection with the entomopathogen Beauveria bassiana. By comparing the metabolites produced by microbes that protect the insect and microbes that cannot protect the insect, the question of how the microbes protect the insect is answered. It turns out that body surface bacteria produce a metabolite cocktail that inhibits colonization of *B.* bassiana and consequently protects the insect. This work reveals novel molecules with antifungal activity, which may aid in discovery and expansion of new prophylactic and therapeutic natural chemicals for treating infectious diseases.

## Abstract (precis)

The protection of associated microbiota for their animal hosts against pathogen infection has been studied widely over the last 100 years. However, how those microbes protect the animal host remains unclear. In former studies, body surface microbes of one insect, Delia antiqua, protected the insect larvae from infection with the entomopathogen Beauveria bassiana. By comparing the metabolites produced by microbes that protect the insect and microbes that cannot protect the insect, the question of how the microbes protect the insect is answered. It turns out that body surface bacteria produce a metabolite cocktail that inhibits colonization of *B.* bassiana and consequently protects the insect. This work reveals novel molecules with antifungal activity, which may aid in discovery and expansion of new prophylactic and therapeutic natural chemicals for treating infectious diseases.

## INTRODUCTION

Microbes form close symbioses with animals (1). Some microbes improve animal fitness such as by protecting their hosts against pathogens (2) and regulating host development (3, 4), while other microbes are lethal pathogens causing severe diseases (5). Studies on deciphering interactions between animals and their beneficial and detrimental microbes have revealed the process of colonization resistance, i.e., the protective effects of the associated microbes against microbial pathogens. For example, indigenous microbiota suppresses resident pathogenic species such as Clostridium difficile to low levels within the intestine (6). Most studies have focused on symbioses of mammals and microbes, revealing the interaction between indigenous microbiota and migratory pathogens during the process of colonization resistance and even identifying various metabolites that mediate the process. These findings likely represent the tip of the iceberg, and such symbioses thus need further investigation.

Colonization resistance in symbioses formed by microbes and insects such as wood wasps (7), bumble bees (8), and beetles (9) has also attracted the attention of ecologists and entomologists. For example, a mixture of four Lactobacillus kunkeei strains isolated from the gut microbial community of bees decreased honeybee infections by the pathogens *Paenibacillus* sp. and Nosema ceranae (10). Microbial probiotic treatment can rescue honeybees from adverse effects due to *N. ceranae* by stimulating immunity in the honeybee (11). Due to insects’ worldwide distribution, rich diversity, the possibility of extrapolating research studies to vertebrates, and the relatively low cost of rearing, insect-microbe symbiotic systems have potential as alternative models to investigate and develop colonization resistance theory in broader taxonomic clades.

Delia antiqua (Meigen) (Diptera: Anthomyiidae) is a devastating pest that feeds on liliaceous crops (12) and can cause a reduction of crop yields by more than half of the total yield (13, 14). Previously, we found that six most frequently isolated bacterial symbionts including Citrobacter freundii, Enterobacter ludwigii, Pseudomonas protegens, Serratia plymuthica, Sphingobacterium faecium, and Stenotrophomonas maltophilia protected *D. antiqua* larvae from Beauveria bassiana infection by inhibiting conidial germination and mycelial growth (15), while the bacterium Klebsiella oxytoca showed no antifungal activity against *B.* bassiana. Collectively, the *D. antiqua*-associated bacterium-entomopathogen tripartite interaction system provides an ideal model to investigate whether and which metabolites produced by the bacterial associates prevent fungal infection of *D. antiqua* larvae.

In this study, we first verified and compared the effects of six bacterial strains including C. freundii B505, *E. ludwigii* B424, P. protegens B108, *S. plymuthica* B585, S. maltophilia B263, and *S. faecium* B253 and one control strain, K. oxytoca B313, on *B.* bassiana and its infection of *D. antiqua* larvae individually. In addition, the effects of the selected bacterial strains on the conidial germination and mycelial growth of *B.* bassiana were also determined. Second, the metabolomic profiles of the six bacterial strains and K. oxytoca B313 were compared to identify candidate metabolites that may protect *D. antiqua* larvae from *B.* bassiana infection. Third, candidate *in situ* metabolites of nonaxenic and axenic larvae were quantified and compared. Fourth, effects of artificial metabolite cocktails (prepared according to *in situ* concentrations of each metabolite) on *B.* bassiana and its infection of *D. antiqua* larvae were determined. This work promotes the understanding of metabolic interactions between entomopathogens and the symbiotic system formed by *D. antiqua* larvae and its associated bacteria. In addition, this work may also provide novel strategies for discovering new natural antifungal metabolites.

## RESULTS

### Experiment I: the six bacterial strains protect *D. antiqua* larvae from fungal infection by inhibiting fungal conidial germination and mycelial growth.

Kaplan-Meier analysis showed that the six bacterial strains including *S. faecium* B253 (see Fig. S1a in the supplemental material, χ^2^ = 0.516, df = 1, *P* = 0.473), *E. ludwigii* B424 (Fig. S1b, χ^2^ = 0.942, df = 1, *P* = 0.334), *S. plymuthica* B585 (Fig. S1c, χ^2^ = 1.440, df = 1, *P* = 0.230), P. protegens B108 (Fig. S1d, χ^2^ = 0.875, df = 1, *P* = 0.350), C. freundii B505 (Fig. S1e, χ^2^ = 0.165, df = 1, *P* = 0.684), and S. maltophilia B263 (Fig. S1f, χ^2^ = 0.406, df = 1, *P* = 0.524) had no significant effects on the survival of axenic larvae compared to the control group. Also, K. oxytoca B313 had no significant effects on the survival of axenic larvae compared to the control group (Fig. S1g, χ^2^ = 0.00407, df = 1, *P* = 0.984).

10.1128/mSystems.00778-20.2 FIG S1 Survival of axenic *D. antiqua* second-instar larvae under the effects of the six selected strains (P. protegens B108 [B108], *S. faecium* B253 [B253], S. maltophilia B263 [B263], *E. ludwigii* B424 [B424], C. freundii B505 [B505], and *S. plymuthica* B585 [B585]) and K. oxytoca B313. The larval survival was estimated with Kaplan-Meier analysis (*n* = 40, one representative experiment of three, log rank test, α = 0.05). “Control” refers to axenic larvae without treatments, and text such as “B505” refers to bacterial strains. Download FIG S1, TIF file, 0.2 MB. Copyright © 2020 Zhou et al. 2020 Zhou et al. This content is distributed under the terms of the Creative Commons Attribution 4.0 International license.

**FIG 1:**
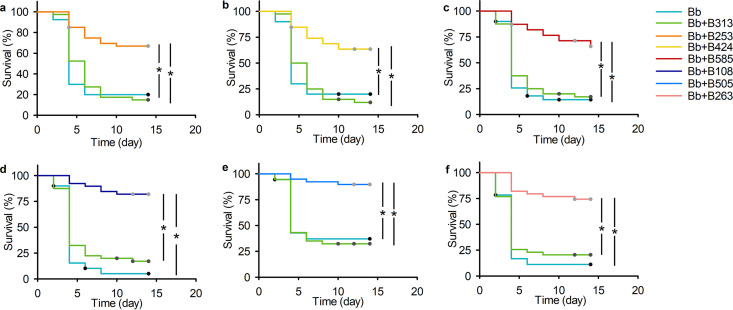
Survival comparison of axenic *D. antiqua* larvae inoculated with the six selected bacterial strains and K. oxytoca B313 under the treatment of *B.* bassiana BB1101. (a) *S. faecium* B253 versus K. oxytoca B313; (b) *E. ludwigii* B424 versus K. oxytoca B313; (c) *S. plymuthica* B585 versus K. oxytoca B313; (d) P. protegens B108 versus K. oxytoca B313; (e) C. freundii B505 versus K. oxytoca B313; (f) S. maltophilia B263 versus K. oxytoca B313. Larval survival was estimated with Kaplan-Meier analysis (*n* = 45, log rank test, α = 0.05). “*” denotes a significant difference between the two connected lines (*P* < 0.05). “Bb” refers to axenic larvae treated with *B.* bassiana, and “Bb+bacterial strain” refers to axenic larvae treated with both *B.* bassiana and the corresponding bacterial strain (*n* = 45/group, one representative experiment of three).

Multiple comparisons were conducted to compare the effect of each selected bacterial strain and K. oxytoca B313 on *B.* bassiana BB1101 infection of *D. antiqua* larvae. Results showed that compared to *B.* bassiana BB1101-treated axenic larvae in each comparison in Fig. 1, K. oxytoca B313 had no effect on *B.* bassiana BB1101-treated larval survival (Fig. 1a, χ^2^ = 0.356, df = 1, *P* = 0.551; Fig. 1b, χ^2^ = 0.177, df = 1, *P* = 0.674; Fig. 1c, χ^2^ = 0.273, df = 1, *P* = 0.601; Fig. 1d, χ^2^ = 1.910, df = 1, *P* = 0.167; Fig. 1e, χ^2^ = 0.109, df = 1, *P* = 0.741; Fig. 1f, χ^2^ = 0.571, df = 1, *P* = 0.450). However, the six selected bacteria showed excellent protective effects for *D. antiqua* larvae against *B.* bassiana BB1101 infection. Specifically, *S. faecium* B253 (Fig. 1a, χ^2^ = 22.676, df = 1, *P* < 0.001), *E. ludwigii* B424 (Fig. 1b, χ^2^ = 20.682, df = 1, *P* < 0.001), *S. plymuthica* B585 (Fig. 1c, χ^2^ = 25.680, df = 1, *P* < 0.001), P. protegens B108 (Fig. 1d, χ^2^ = 54.655, df = 1, *P* < 0.001), C. freundii B505 (Fig. 1e, χ^2^ = 23.478, df = 1, *P* < 0.001), and S. maltophilia B263 (Fig. 1f, χ^2^ = 36.165, df = 1, *P* < 0.001) significantly increased the survival of *B.* bassiana-treated axenic larvae compared to control (Bb). For each set of the comparison (Fig. 1), the survival of *B.* bassiana-treated axenic larvae inoculated with one of the six bacterial strains was significantly higher than that of K. oxytoca B313 (B253, χ^2^ = 23.901, df = 1, *P* < 0.001; B424, χ^2^ = 24.779, df = 1, *P* < 0.001; B585, χ^2^ = 25.680, df = 1, *P* < 0.001; B108, χ^2^ = 37.920, df = 1, *P* < 0.001; B505, χ^2^ = 27.977, df = 1, *P* < 0.001; B263, χ^2^ = 26.614, df = 1, *P* < 0.001). Specifically, survival rates of *B.* bassiana-treated axenic larvae inoculated with *S. faecium* B253, *E. ludwigii* B424, *S. plymuthica* B585, P. protegens B108, C. freundii B505, and S. maltophilia B263 were 67.5%, 64.1%, 66.7%, 82.1%, 89.7%, and 74.4%, respectively, and survival rates of *B.* bassiana-treated axenic larvae inoculated with of K. oxytoca B313 in each corresponding comparison were 15.0%, 12.5%, 17.5%, 17.5%, 32.4%, and 20.5%, respectively.

All six selected bacterial strains showed significant inhibition of conidial germination of *B.* bassiana BB1101 in a dose-dependent manner (Fig. S2a; B253, one-way analysis of variance [ANOVA], F_3,20_ = 1,510.668, *P* < 0.001; B424, Welch’s ANOVA, F_3,10.525_ = 7,763.650, *P* < 0.001; B585, Welch’s ANOVA, F_3,10.634_ = 2,020.052, *P* < 0.001; B108, Welch’s ANOVA, F_3,10.327_ = 4,219.258, *P* < 0.001; B505, Welch’s ANOVA, F_3,9.541_ = 1,375.56, *P* < 0.001; B263, one-way ANOVA, F_3,20_ = 1,255.472, *P* < 0.001), while K. oxytoca B313 did not affect conidial germination (Fig. S2a, one-way ANOVA, F_3,20_ = 1.427, *P* = 0.264). Specifically, conidial germination of *B.* bassiana BB1101 was decreased to 24.8% to 2.9% by supernatants of the six bacterial cultures. Similarly, the six bacterial strains significantly inhibited the mycelial growth of *B.* bassiana BB1101 in a dose-dependent manner (Fig. S2b; B253, one-way ANOVA, F_3,20_ = 758.197, *P* < 0.001; B424, Welch’s ANOVA, F_3,9.668_ = 1,285.678, *P* < 0.001; B585, Welch’s ANOVA, F_3,9.296_ = 1,434.239, *P* < 0.001; B108, Welch’s ANOVA, F_3,9.721_ = 1,288.682, *P* < 0.001; B505, Welch’s ANOVA, F_3,10.283_ = 1,137.907, *P* < 0.001; B263, Welch’s ANOVA, F_3,10.199_ = 1,218.094, *P* < 0.001), while K. oxytoca B313 did not affect mycelial growth (Fig. S2b, one-way ANOVA, F_3,20_ = 0.940, *P* = 0.440). Specifically, mycelial growth of *B.* bassiana BB1101 was reduced by up to 90% compared to the control under the dose of 500 CFU/petri dish by each of the six selected bacterial strains.

10.1128/mSystems.00778-20.3 FIG S2 Conidial germination (a) and mycelial growth (b) of *B.* bassiana BB1101 under the effect of the selected six strains (P. protegens B108 [B108], *S. faecium* B253 [B253], S. maltophilia B263 [B263], *E. ludwigii* B424 [B424], C. freundii B505 [B505], and *S. plymuthica* B585 [B585]) and K. oxytoca B313 (B313). Values for each bar plot represent the conidial germination and mycelial growth rate relative to the control (± SD). Different letters above each bar refer to significant differences of multiple comparisons by the Tukey method within each set of bars (*n* = 6, “Control” = LB media in panel a, and “Control” = 0 CFU/petri dish in panel b). Download FIG S2, TIF file, 0.2 MB. Copyright © 2020 Zhou et al. 2020 Zhou et al. This content is distributed under the terms of the Creative Commons Attribution 4.0 International license.

### Experiment II: metabolomic analysis revealed 10 candidate metabolites from the six bacterial strains that may protect *D. antiqua* larvae.

In total, 17,983 and 12,275 metabolites were detected in all samples under positive and negative ion modes, respectively. Annotations were made for 6,389 metabolites among 14,522 metabolites with high-quality features under positive mode and 3,688 metabolites among 10,476 metabolites with high-quality features under negative mode. The unsupervised model principal-component analysis (PCA) showed that the samples from each group including quality control (QC) showed a trend of shifting away from each other under both positive (Fig. S3a) and negative (Fig. S3b) ion modes.

10.1128/mSystems.00778-20.4 FIG S3 PCA score plots for metabolomic samples from the selected six strains (P. protegens B108 [B108], *S. faecium* B253 [B253], S. maltophilia B263 [B263], *E. ludwigii* B424 [B424], C. freundii B505 [B505], and *S. plymuthica* B585 [B585]), K. oxytoca B313 (B313), LB medium (LB), and quality control samples (QC) in positive (a) and negative (b) modes. Download FIG S3, TIF file, 1.2 MB. Copyright © 2020 Zhou et al. 2020 Zhou et al. This content is distributed under the terms of the Creative Commons Attribution 4.0 International license.

To compare the differences in metabolites between lysogeny broth (LB) medium and each of the bacterial strains, individual PCAs and partial least-squares discriminant analyses (PLS-DAs) (Fig. S4) were conducted. PCA results showed that samples from selected bacterial strains and LB media were clearly separated from each other under both positive and negative ion modes. Similar separating trends were also observed in PLS-DA (Fig. S4). For all permutation tests of PLS-DA models of selected bacterial strains and LB medium, all blue Q2 values to the left are lower than the original points to the right, and the regression line of the blue Q2 points intersects the vertical axis below zero, which indicated a low risk of model overfitting (data not shown).

10.1128/mSystems.00778-20.5 FIG S4 Individual PCA and PLS-DA score plots for metabolomic samples of selected bacterial strains and K. oxytoca B313 (B313) against that from LB medium (LB) in positive and negative modes. a1 to g1: PCA for P. protegens B108 (B108) versus LB, *S. faecium* B253 (B253) versus LB, S. maltophilia B263 (B263) versus LB, K. oxytoca B313 (B313) versus LB, *E. ludwigii* B424 (B424) versus LB, C. freundii B505 (B505) versus LB, and *S. plymuthica* B585 (B585) versus LB, respectively, under positive modes. a2 to g2: PCA for P. protegens B108 (B108) versus LB, *S. faecium* B253 (B253) versus LB, S. maltophilia B263 (B263) versus LB, K. oxytoca B313 (B313) versus LB, *E. ludwigii* B424 (B424) versus LB, C. freundii B505 (B505) versus LB, and *S. plymuthica* B585 (B585) versus LB, respectively, under negative modes. a3 to g3: PLS-DA for P. protegens B108 (B108) versus LB, *S. faecium* B253 (B253) versus LB, S. maltophilia B263 (B263) versus LB, K. oxytoca B313 (B313) versus LB, *E. ludwigii* B424 (B424) versus LB, C. freundii B505 (B505) versus LB, and *S. plymuthica* B585 (B585) versus LB, respectively, under positive modes. a4 to g4: PLS-DA for P. protegens B108 (B108) versus LB, *S. faecium* B253 (B253) versus LB, S. maltophilia B263 (B263) versus LB, K. oxytoca B313 (B313) versus LB, *E. ludwigii* B424 (B424) versus LB, C. freundii B505 (B505) versus LB, and *S. plymuthica* B585 (B585) versus LB, respectively, under negative modes. Download FIG S4, TIF file, 1.4 MB. Copyright © 2020 Zhou et al. 2020 Zhou et al. This content is distributed under the terms of the Creative Commons Attribution 4.0 International license.

**FIG 2:**
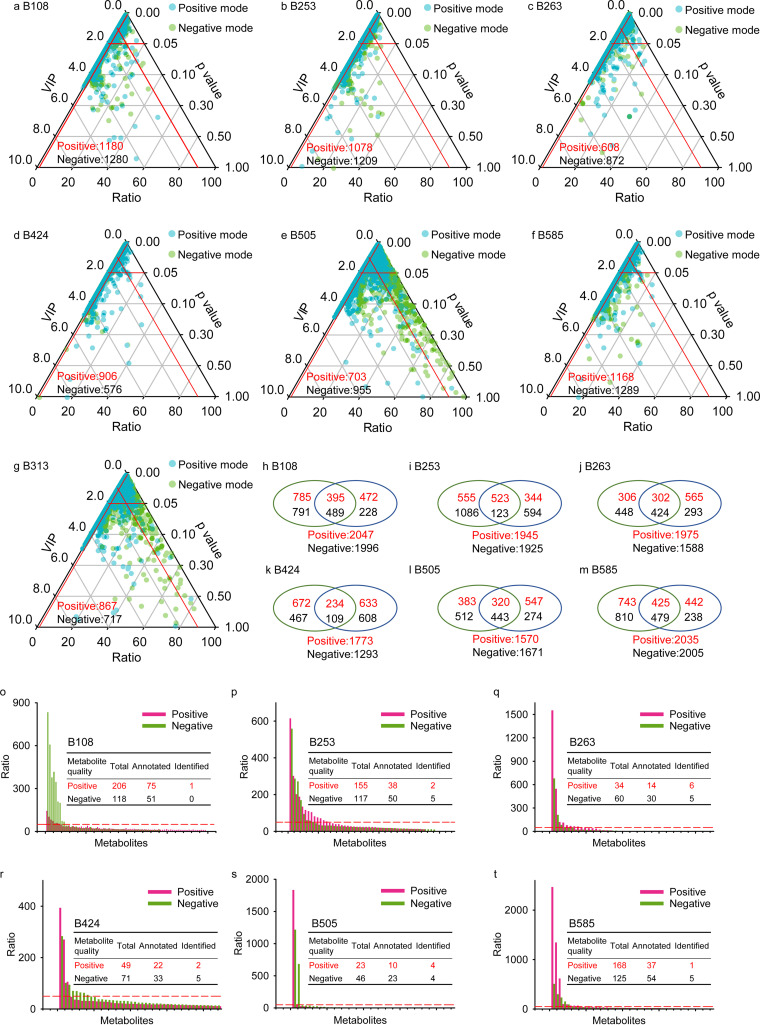
Candidate metabolite screening process. (a to g) Metabolites produced by bacterial strains B108, B253, B263, B424, B505, B585, and B313 compared to LB, respectively (inside the small red triangle). (h to m) Data set unions created by merging metabolites from B313 with B108, B253, B263, B424, B505, and B585, respectively. (o to t) Metabolite abundance upregulated ratios of B108, B253, B263, B424, B505, and B585 compared with B313, respectively.

There were 1,180, 1,078, 608, 906, 703, 1,168, and 867 metabolites under positive mode and 1,280, 1,209, 872, 576, 955, 1,289, and 717 metabolites under negative mode screened as bacterium-derived metabolites compared to LB medium for B108, B253, B263, B424, B505, B585, and B313, respectively (Fig. 2a to g). The unions of each selected strain (including B108, B253, B263, B424, B505, and B585) with B313 were obtained. In total, 2,047, 1,945, 1,975, 1,773, 1,570, and 2,035 metabolites under positive mode and 1,996, 1,925, 1,588, 1,293, 1,671, and 2,005 metabolites under negative mode were obtained, respectively, for the union of B313 and each selected strain (B108, B253, B263, B424, B505, and B585, Fig. 2h to m). Among those unified data sets, 206, 155, 34, 49, 23, and 168 metabolites under positive mode and 118, 117, 60, 71, 46, and 125 metabolites under negative mode were obtained for B108, B253, B263, B424, B505, and B585, respectively, with relative abundances of 10 times that of B313 (Fig. 2o to t).

Only some of those metabolites were annotated (75, 38, 14, 22, 10, and 37 metabolites under positive mode and 51, 50, 30, 33, 23, and 54 metabolites under negative mode for B108, B253, B263, B424, B505, and B585, respectively), and 17 metabolites under positive mode and 24 metabolites under negative mode were ultimately identified (1, 2, 6, 2, 4, and 1 metabolite under positive mode and 0, 5, 5, 5, 4, and 5 metabolites under negative mode for B108, B253, B263, B424, B505, and B585, respectively). Among those metabolites, l-tyrosine, l-sorbose, l-leucine, and l-valine are common compounds usually used by microorganisms as nutrients. Thus, those chemicals were excluded for further anti-*B.* bassiana bioassay. The other 10 chemicals, including glutaric acid, thymine, ethylmalonic acid, hypoxanthine, kynurenic acid, picolinic acid, ketoisocaproic acid, phenyllactic acid, adipic acid, and indoleacetic acid (corresponding quantification and detailed identification information are in Fig. S5), were regarded as candidate metabolites that may protect *D. antiqua* larvae from *B.* bassiana BB1101 infection (Table 1).

**TABLE 1:** Candidate bacterial metabolites from selected bacterial strains

No.	Metabolite ID	Retention time (s)	*m/z*	Detection mode	Ratio	*P* value	VIP	Sample	Candidate metabolite	CAS no.
1	M190T192	192	190	Positive	84.57	1.50E−05	2.51	P. protegens B108	Kynurenic acid	492-27-3


2	M115T124	124	115	Positive	116.47	4.55E−08	3.02	*S. faecium* B253	Ethylmalonic acid	601-75-2
3	M131T125	125	131	Negative	272.19	2.05E−09	3.82		Glutaric acid	110-94-1
4	M145T115	115	145	Negative	35.59	6.98E−13	3.08		Adipic acid	124-04-9
5	M125T103	103	125	Negative	16.01	9.73E−07	2.27		Thymine	65-71-4


6	M124T65	65	124	Positive	1,552.84	4.14E−06	6.40	S. maltophilia B263	Picolinic acid	98-98-6
7	M124T82	82	124	Positive	546.33	5.32E−09	5.57		Picolinic acid	98-98-6
8	M137T63	63	137	Positive	96.63	1.00E−10	4.74		Hypoxanthine	68-94-0
9	M115T124	124	115	Positive	79.63	7.77E−08	4.69		Ethylmalonic acid	601-75-2
10	M176T307	307	176	Positive	54.29	6.79E−17	3.65		Indoleacetic acid	87-51-4
11	M137T84	84	137	Positive	40.63	4.43E−07	3.83		Hypoxanthine	68-94-0
12	M135T84	84	135	Negative	379.16	1.45E−07	5.41		Hypoxanthine	68-94-0
13	M131T125	125	131	Negative	211.67	5.44E−09	4.61		Glutaric acid	110-94-1
14	M145T115	115	145	Negative	27.01	1.04E−11	3.58		Adipic acid	124-04-9
15	M125T103	103	125	Negative	17.56	3.51E−06	2.97		Thymine	65-71-4


16	M124T65	65	124	Positive	1,834.90	3.82E−06	6.00	C. freundii B505	Picolinic acid	98-98-6
17	M137T63	63	137	Positive	102.63	1.31E−10	4.41		Hypoxanthine	68-94-0
18	M190T192	192	190	Positive	40.13	3.30E−05	3.49		Kynurenic acid	492-27-3
19	M137T84	84	137	Positive	39.37	1.87E−07	3.54		Hypoxanthine	68-94-0
20	M135T84	84	135	Negative	385.13	1.33E−07	4.96		Hypoxanthine	68-94-0
21	M131T125	125	131	Negative	26.74	2.47E−08	2.59		Glutaric acid	110-94-1
22	M125T103	103	125	Negative	18.24	2.13E−06	2.75		Thymine	65-71-4
23	M145T115	115	145	Negative	12.45	2.34E−11	2.51		Adipic acid	124-04-9


24	M115T124	124	115	Positive	99.99	9.33E−09	2.83	*S. plymuthica* B585	Ethylmalonic acid	601-75-2
25	M131T125	125	131	Negative	233.92	6.66E−10	3.67		Glutaric acid	110-94-1
26	M145T115	115	145	Negative	40.09	9.46E−13	3.14		Adipic acid	124-04-9
27	M125T103	103	125	Negative	14.71	2.48E−06	2.18		Thymine	65-71-4


28	M124T226	226	124	Positive	270.36	1.01E−06	4.35	*E. ludwigii* B424	Picolinic acid	98-98-6
29	M129T65	65	129	Negative	35.08	2.01E−06	3.70		Ketoisocaproic acid	816-66-0
30	M129T41	41	129	Negative	18.13	8.04E−05	3.36		Ketoisocaproic acid	816-66-0
31	M165T154	154	165	Negative	15.91	8.20E−07	3.22		Phenyllactic acid	828-01-3
32	M165T106	106	165	Negative	12.30	5.67E−12	3.08		Phenyllactic acid	828-01-3

10.1128/mSystems.00778-20.6 FIG S5 Extracted ion chromatogram (1) and matched peaks to the in-house fragment spectrum library (2). (a) M190T192; (b) M115T124; (c) M131T125; (d) M145T115; (e) M125T103; (f) M124T65; (g) M124T82; (h) M137T63; (i) M176T307; (j) M137T84; (k) M135T84; (l) M124T226; (m) M129T65; (n) M129T41; (o) M165T154; (p) M165T106. Download FIG S5, TIF file, 1.6 MB. Copyright © 2020 Zhou et al. 2020 Zhou et al. This content is distributed under the terms of the Creative Commons Attribution 4.0 International license.

### Experiment III: concentrations of candidate metabolites on the body surface of axenic and nonaxenic *D. antiqua* larvae were different.

**FIG 3:**
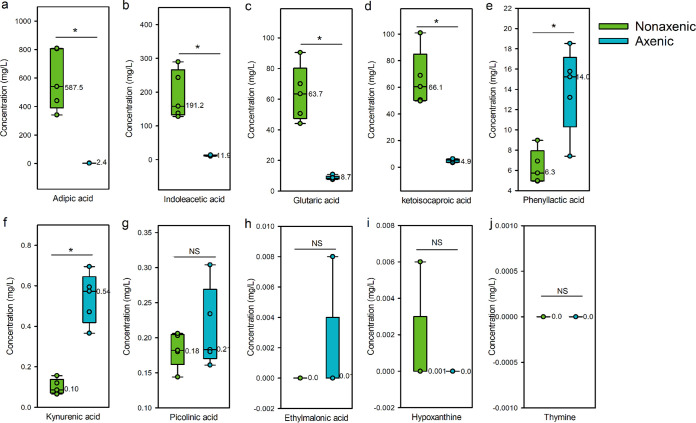
Concentrations of 10 candidate metabolites on the body surface of axenic and nonaxenic *D. antiqua* larvae. (a to j) Adipic acid, indoleacetic acid, glutaric acid, ketoisocaproic acid, phenyllactic acid, kynurenic acid, picolinic acid, ethylmalonic acid, hypoxanthine, and thymine, respectively. “*” denotes a significant difference between the two boxes (*P* < 0.05), and “NS” denotes no significant difference between the two boxes (*P* > 0.05). Small circles inside the box refer to the original value for each test, and numerical labels near the boxes refer to the mean of the original value (*n* = 5).

For the 10 candidate metabolites on the body surface of *D. antiqua* larvae (Fig. 3a to j), nine were detected on either nonaxenic larvae or axenic larvae, while thymine was not detected on nonaxenic larvae or axenic larvae. Moreover, the quantities of the nine metabolites on nonaxenic larvae varied from those on axenic larvae. Specifically, the concentrations of adipic acid (Fig. 3a, independent samples *t* test, df = 4.000, *t* = 6.153, *P* = 0.004), indoleacetic acid (Fig. 3b, independent samples *t* test, df = 4.040, *t* = 5.618, *P* = 0.005), glutaric acid (Fig. 3c, independent samples *t* test, df = 4.003, *t* = 6.673, *P* = 0.002), and ketoisocaproic acid (Fig. 3d, independent samples *t* test, df = 4.028, *t* = 6.532, *P* = 0.003) on nonaxenic larval body surface were 587.5, 191.2, 63.7, and 66.1 mg/liter, respectively, which were significantly higher than those of axenic larval body surface (2.4, 11.9, 8.7, and 4.9 mg/liter, respectively). In contrast, the concentrations of phenyllactic acid (Fig. 3e, independent samples *t* test, df = 5.283, *t* = −3.481, *P* < 0.05) and kynurenic acid (Fig. 3f, independent samples *t* test, df = 8, *t* = −7.499, *P* = 0.0001) on nonaxenic larval body surface were 6.3 and 0.1 mg/liter, respectively, which were significantly lower than those of axenic larval body surface (14.0 and 0.54 mg/liter, respectively). In addition, the concentrations of picolinic acid (Fig. 3g, independent samples *t* test, df = 8, *t* = −1.044, *P* = 0.327), ethylmalonic acid (Fig. 3h, Mann-Whitney U test, U = 10, *z* = −1, *P* = 0.690), and hypoxanthine (Fig. 3i, Mann-Whitney U test, U = 10, *z* = −1, *P* = 0.690) were 0.18, 0.0, and 0.001 mg/liter, respectively, which were not significantly different from those of the axenic larval body surface (0.21, 0.01, and 0.0 mg/liter, respectively).

### Experiment IV: bacterial metabolite cocktail of nonaxenic *D. antiqua* larvae inhibited the conidial germination and mycelial growth of *B.* bassiana BB1101.

**FIG 4:**
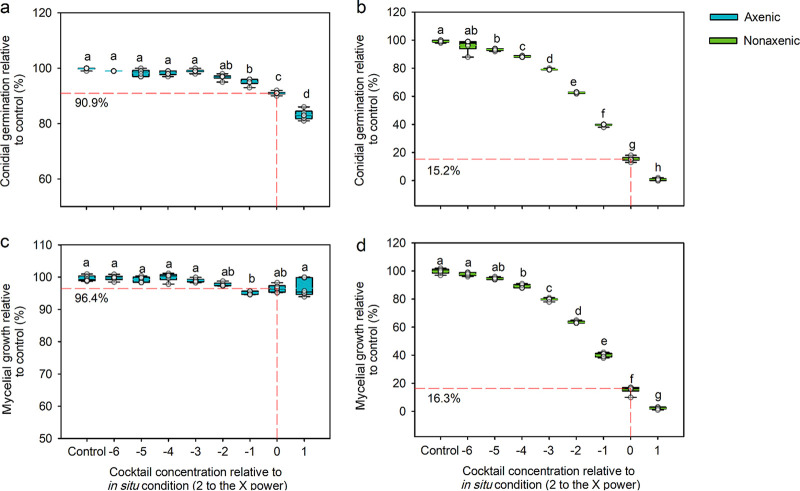
Conidial germination and mycelial growth of *B.* bassiana BB1101 in the presence of two metabolite cocktails. (a and b) Conidial germination of *B.* bassiana BB1101 under the effect of a series of concentrations of metabolite cocktails from the body surface of axenic and nonaxenic larvae, respectively. (c and d) Mycelial growth of *B.* bassiana BB1101 under the effect of a series of concentrations of metabolite cocktails from the body surface of axenic and nonaxenic larvae, respectively. Values in the box plots represent the conidial germination rate or mycelial growth rate relative to the control (± SD). Different letters above each box indicate significant differences of multiple comparisons within each set of boxes (*n* = 6; “Control” = 0 mg/liter).

Results showed that metabolite cocktails for the body surfaces of axenic larvae (Fig. 4a, one-way ANOVA, F_8,45_ = 185.151, *P* < 0.001) and nonaxenic larvae (Fig. 4b, Welch’s ANOVA, F_8,18.484_ = 11,351.398, *P* < 0.001) significantly inhibited conidial germination of *B.* bassiana BB1101 individually in a dose-dependent manner. As the concentration of each individual cocktail increased, the conidial germination decreased significantly (Fig. 4). Specifically, under the effect of metabolite cocktail for axenic larval body surface at a dosage equal to *in situ* conditions, conidial germination of *B.* bassiana BB1101 was slightly reduced to 90.9% relative to control (Fig. 4a), while conidial germination of *B.* bassiana BB1101 was sharply reduced to 15.2% relative to control under the effect of metabolite cocktail for nonaxenic larval body surface at a dosage equal to *in situ* conditions (Fig. 4b).

Similar inhibitory effects of the two metabolite cocktails on the mycelial growth of *B.* bassiana BB1101 were detected. Results showed that metabolite cocktails for body surfaces of axenic larvae (Fig. 4c, Welch’s ANOVA, F_8,18.605_ = 32.641, *P* < 0.001) and nonaxenic larvae (Fig. 4d, one-way ANOVA, F_8,45_ = 7,772.135, *P* < 0.001) each significantly inhibited mycelial growth of *B.* bassiana BB1101 individually. As the concentrations of metabolite cocktails for body surface of nonaxenic larvae increased, the mycelial growth decreased. Under the effect of a metabolite cocktail for axenic larval body surface at a dosage equal to the *in situ* conditions, mycelial growth of *B.* bassiana BB1101 was slightly reduced to 96.4% relative to control (Fig. 4c), while for nonaxenic larval body surface mycelial growth of *B.* bassiana BB1101 was significantly reduced to 16.3% relative to control under the effect of a metabolite cocktail at a dosage equal to *in situ* conditions (Fig. 4d).

### Experiment V: artificial metabolite cocktail for nonaxenic larval body surface inhibited fungal infection of *D. antiqua* larvae with *B.* bassiana BB1101.

**FIG 5:**
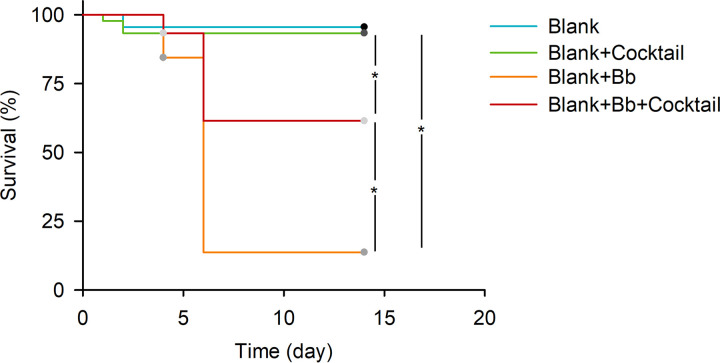
Survival of *B.* bassiana-treated axenic *D. antiqua* larvae in the presence of the metabolite cocktail for nonaxenic larval body surface at the dosage equal to *in situ* conditions. Blue lines: axenic larvae without microbial or cocktail treatments (Blank). Green lines: axenic larvae treated with the metabolite cocktail for nonaxenic larval body surface at the dosage equal to *in situ* conditions (Blank+Cocktail). Orange lines: axenic larvae treated with conidial suspension of *B.* bassiana BB1101 (Blank+Bb). Red lines: axenic larvae treated with the metabolite cocktail for nonaxenic larval body surface at the dosage equal to *in situ* conditions as well as the conidial suspension of *B.* bassiana BB1101 (Blank+Bb+Cocktail). Larval survival was estimated with Kaplan-Meier analysis (*n* = 45, log rank test, α = 0.05). “*” denotes a significant difference between the two connected lines (*P* < 0.05) (*n* = 45/group, one representative experiment of three).

Kaplan-Meier analysis showed that the *B.* bassiana BB1101 treatment significantly decreased the survival of axenic larvae (Fig. 5, log rank test, χ^2^ = 52.755, df = 1, *P* < 0.001), while the metabolite cocktail showed no effects on the survival of axenic *D. antiqua* larvae (Fig. 5, log rank test, χ^2^ = 0.225, df = 1, *P* = 0.636). For the *B.* bassiana-infected axenic larvae, the final survival of larvae that were preliminarily treated with the metabolite cocktail was significantly higher than that of the larvae without the metabolite cocktail treatment (Fig. 5, χ^2^ = 19.428, df = 1, *P* < 0.001), though the cocktail cannot exclusively prevent mortality. Specifically, under *B.* bassiana BB1101 treatment, the survival rate of axenic larvae treated with the metabolite cocktail was 62.2%, while the survival rate of axenic larvae that were not treated with the candidate metabolite cocktail was 15.6%.

## DISCUSSION

The symbiotic system formed by bacteria and *D. antiqua* larvae provides an ideal model for studying the process of colonization resistance and identifying metabolites that mediate the process. In this study, six most frequently isolated species (C. freundii, *E. ludwigii*, P. protegens, *S. plymuthica*, S. maltophilia, and *S. faecium*) and K. oxytoca B313 showed different effects on the infection of *D. antiqua* larvae with *B.* bassiana. Specifically, neither the six bacterial strains nor K. oxytoca B313 affected the survival of axenic *D. antiqua* larvae (see Fig. S1 in the supplemental material). However, the six bacterial strains inhibited *B.* bassiana infection of axenic *D. antiqua* larvae, while K. oxytoca B313 did not (Fig. 1). We confirmed that the six bacterial strains protect *D. antiqua* larvae by inhibiting the conidial germination (Fig. S2a) and mycelial growth (Fig. S2b) of *B.* bassiana, which is consistent with previous reports (15). Comparison of metabolomic data from the six bacterial strains and K. oxytoca B313 (Fig. 2) indicated that 10 types of metabolites, including glutaric acid, thymine, ethylmalonic acid, hypoxanthine, kynurenic acid, picolinic acid, ketoisocaproic acid, phenyllactic acid, adipic acid, and indoleacetic acid, were produced at different levels among the six bacteria compared to B313. These metabolites may be the bioactive chemicals that protect *D. antiqua* larvae from entomopathogenic infection.

Subsequent experiments quantified these 10 chemicals on the body surface of both axenic and nonaxenic *D. antiqua* larvae (Fig. 3). According to the *in situ* concentration of each chemical from samples of axenic and nonaxenic larval body surfaces, two kinds of metabolite cocktails were prepared in a series of concentrations. Further experiments revealed that the metabolite cocktail of the axenic larval body surface showed a slightly inhibitory effect on both conidial germination (Fig. 4a) and mycelial growth (Fig. 4c) of *B.* bassiana BB1101 at a dosage equal to *in situ* conditions, while the metabolite cocktail of the nonaxenic larval body surface showed a significantly inhibitory effect on both conidial germination (Fig. 4b) and mycelial growth (Fig. 4d) of *B.* bassiana BB1101 at a dosage equal to *in situ* conditions. Furthermore, *B.* bassiana BB1101’s successful infection of axenic larvae may result from the concentration and chemical composition of the metabolite cocktail, which was confirmed in Fig. 5.

Colonization resistance involves direct interactions between the associated microbiota and the pathogens (16). The six bacteria associated with *D. antiqua* selected in this study directly inhibited conidial germination and mycelial growth of *B.* bassiana by producing a metabolite cocktail including glutaric acid, ethylmalonic acid, picolinic acid, ketoisocaproic acid, adipic acid, and indoleacetic acid. Indoleacetic acid has been reported to mediate colonization resistance by inhibiting biofilm in mammal guts (17), while the essential roles of glutaric acid, ethylmalonic acid, picolinic acid, ketoisocaproic acid, and adipic acid in mediating colonization resistance were reported for the first time in this study. Antimicrobial activity of these organic acids has been reported occasionally. For example, glutaric acid was found in metabolites of an antifungal *Bacillus* strain (18), and direct evidence of virucidal activity of glutaric acid has also been reported (19). Similarly, antimicrobial activity of picolinic acid (20, 21), adipic acid (22), and indoleacetic acid (23) has also been reported occasionally. It seems that microbes associated with *D. antiqua* mediate colonization resistance against *B.* bassiana by producing anti-*B.* bassiana metabolites.

Associated microbiota mediate colonization resistance by producing antimicrobial metabolites (24), which indicates that the active metabolites should be accumulated *in situ*. Correspondingly, concentrations of glutaric acid, adipic acid, indoleacetic acid, and ketoisocaproic acid from nonaxenic larval body surfaces were much higher than those from axenic ones (Fig. 3). Higher concentrations of certain organic acids on body surfaces of nonaxenic larvae may result from the microbial production of certain organic acids from plant amino acids such as tryptophan, leucine, or lysine as well as several other amino acids that have been detected in garlic and onion (25, 26). For example, glutaric acid is naturally produced during the metabolism of lysine and tryptophan by bacteria (27). Indoleacetic acid is produced by various bacteria through metabolism of tryptophan (28). Ketoisocaproic acid is an intermediate in the metabolism of leucine (29). Taking phylogenetic relationships of the six bacterial species into consideration (15), it seems that these bacteria produce those organic acids mainly through primary metabolization. Those metabolites may be essential in colonization resistance. In contrast, concentrations of phenyllactic acid and kynurenic acid from nonaxenic larval body surfaces were much lower than those from axenic ones (Fig. 3). This may result from the microbial assimilation of certain organic acids from plant tissue leading to reduction of precursors for phenyllactic acid and kynurenic acid on the nonaxenic insects. Even though concentrations of phenyllactic acid and kynurenic acid from axenic larval body surfaces were significantly higher than those from nonaxenic ones, this difference may not influence the whole scenery of colonization resistance as concentrations of phenyllactic acid (Fig. 3e, 6.3 to 14.0 mg/liter) and kynurenic acid (Fig. 3f, 0.1 to 0.5 mg/liter) were much lower than those of other organic acids from nonaxenic larval body surfaces (Fig. 3a, adipic acid, 587.5 mg/liter; Fig. 3b, indoleacetic acid, 191.2 mg/liter; Fig. 3c, glutaric acid, 63.7 mg/liter; Fig. 3d, ketoisocaproic acid, 66.1 mg/liter). Besides, the *in vivo* abundance of the microbes tested in this study in *D. antiqua* remains unclear although they were most frequently isolated species in former studies (15). Dominant species, especially those unculturable ones, in the symbiotic system formed by *D. antiqua* larvae and bacteria may also play a significant role in metabolite transformations, which may explain why the *in vivo* metabolite abundances do not match those predicted from the *in vitro* experiments using individual strains. Further investigation combining microbiome analysis and metabolomic analysis *in vivo* needs to be conducted.

Several reasons may explain the anti-*B.* bassiana activity of the metabolite cocktail. First, the organic acid may disrupt primary metabolomic activity of *B.* bassiana during conidial germination and mycelial growth as most organic acids can cross cell membranes (30, 31). For example, the antimicrobial activity of picolinic acid (a metal-chelating agent) may result from the deprivation of free nutrient iron essential for the conidial germination and mycelial growth of *B.* bassiana, which has been reported previously (21, 32). As an intermediate of the metabolism of leucine (29), ketoisocaproic acid has been reported to suppress insulin-stimulated glucose transport in skeletal muscle cells (33). This metabolite might function in glucose transport in *B.* bassiana, which leads to repressed conidial germination and mycelial growth of the fungus. Second, the accumulation of adipic acid, indoleacetic acid, glutaric acid, and ketoisocaproic acid on nonaxenic *D. antiqua* larval body surface (Fig. 3a to d) may lead to a low-pH condition for the larval body surface as all these compounds belong to organic acids. Although the concentration of phenyllactic acid and kynurenic acid decreased on body surfaces of nonaxenic *D. antiqua* larvae compared to axenic ones, this may scarcely contribute to increase of the lowered-pH condition as the concentrations of phenyllactic acid and kynurenic acid were much lower than those of adipic acid, indoleacetic acid, glutaric acid, and ketoisocaproic acid (Fig. 3). Combined with previous studies showing that low pH (below 6.3) leads to mycelial growth inhibition for *B.* bassiana (34), it seems that the candidate metabolites decreased the pH of the larval habitat and thus protected the larvae from *B.* bassiana infection. Third, low pH of the *D. antiqua* larval habitat may in turn enhance the toxicity of organic acids to the fungus. For example, adipic acid will become undissociated and enter cells via passive diffusion over the plasma membrane. Once in the cytosol, the carboxylic groups of this acid become deprotonated due to the almost neutral pH in the cytosol and cause acid stress in the cell (35). Further experiments such as testing the *B.* bassiana infection of *D. antiqua* after neutralizing the low pH, the transcriptomic response of *B.* bassiana against the organic acid cocktail, and the synergistic effects of individual organic acids need to be conducted to illustrate the molecular mechanism of the protective effect of the organic acid cocktail.

As no standard protocols are available to identify differential metabolites from different bacterial strains that mediate colonization resistance, especially considering the background noise from microbial culture media, we took a relatively stricter cutoff strategy to detect candidate metabolites that mediate colonization resistance in the *D. antiqua*-microbe symbiosis. Based on the hypothesis that the most discrepant metabolites between the six bacterial strains and K. oxytoca B313 may be the chemicals with the highest potential to protect *D. antiqua* larvae from fungal infection, candidate metabolite chemicals were quantified *in situ* and their effects on the fungal infection of *D. antiqua* larvae were determined. Although this design may lead to artificial deviations in detecting acting members among the metabolite pool, especially when detecting potential metabolites with minor differences between the two groups of bacterial strains, several metabolites that mediate colonization resistance were successfully detected. Besides, only a very small portion of metabolites were annotated and identified as shown in Fig. 2. There should be a much greater metabolic diversity of unknown composition and function for the natural situation in the *D. antiqua*-microbe symbiosis. This work focused only on the small subset of metabolites that could be identified by database annotation, for which there is no technological alternative at present.

Moreover, as shown in Fig. 2o to t, the majority of differential metabolites were not annotated or identified. There are likely more metabolites than those selected in this study that provide protection for the insect host. Further experiments to isolate metabolites with chromatographic column separation and identify the bioactivity of these metabolites need to be conducted. Bacterial strains selected in this work are the most frequently isolated bacterial strains among all strains obtained in prior research by a culture-dependent method, which indicates that the selected bacteria in this work may not be the dominant species among the associated microbiota of *D. antiqua*. Thus, our work identified a limited number of metabolites that function in colonization resistance in *D. antiqua*-bacterium symbiosis, consistent with the assumption that identified metabolites that mediate colonization resistance are likely a small proportion of the total (17). Further analysis combining data of the host microbiome and metabolomic data sets may lead to deep understanding of the process of colonization resistance in *D. antiqua*-bacterium symbiosis.

During the process of colonization resistance, associated microbiota can kill other microbes by secreting small molecules such as short-chain fatty acids and small carboxylic acids as well as some tryptophan metabolites (17) in mammal guts. For insects, some metabolites mediating colonization resistance are some antibiotics. For example, the *Penicillium* symbionts associated with a leaf-rolling weevil defend the weevil’s offspring from mold fungi and antagonistic bacteria by producing the antibiotic (+)-scleroderolide (36). *Streptomyces* microbial symbionts associated with beewolves can produce antibiotics including piericidin derivatives, streptochlorin derivatives, and nigericin to protect the insect host against mold fungi (7). However, in the symbiotic system formed by the *D. antiqua* larvae and their associated bacteria, the metabolite cocktail of organic acids derived from amino acids (excluding tryptophan) has been proven to protect the insect host from *B.* bassiana infection. Their functions in mediating colonization resistance were reported for the first time. Given the essential roles of those novel metabolites that mediate colonization resistance, this work may aid in discovery and expansion of the list of new bioactive antibiotics, promoting development of prophylactic and therapeutic approaches for treating many infectious diseases for both humans and other animals.

## MATERIALS AND METHODS

### Insects, microbial strains, and materials.

Adults of *D. antiqua* were originally collected from garlic fields in Taian City, China (N36°14′, E117°25′). As this insect is not in the list of wild animals under state priority conservation in China, permissions to collect it were not needed. Eggs laid by these field-collected adult females were randomly selected for further experiments. Axenic larvae were obtained by rearing larvae from hatched surface-sterilized eggs (75% ethanol twice for 30 s) with axenic artificial diets containing antibiotics (12, 15). Detailed information on the preparation of artificial diets is in Table S1 in the supplemental material. Axenic second-instar larvae (hatched larvae reared with artificial diets at 24°C for 7 days in darkness) were used in this study as their body size is suitable for experimental procedures and there was enough time for experimental observation before pupation. Additional bacterial and fungal isolation experiments from axenic larvae (15) were conducted to ensure that all culturable microbes were eliminated in the axenic larvae. Nonaxenic second-instar larvae were collected from garlic fields. The fungus *B.* bassiana BB1101 was originally isolated from infected *D. antiqua* adults with the single-spore method (37), and its conidia were preserved in 25% glycerol in our laboratory at −80°C. The bacterial strains C. freundii B505 (GenBank accession no. MF084973), *E. ludwigii* B424 (GenBank accession no. MF084966), P. protegens B108 (GenBank accession no. MF084946), *S. plymuthica* B585 (GenBank accession no. MF084977), S. maltophilia B263 (GenBank accession no. MF084954), and *S. faecium* B253 (GenBank accession no. MF084952), which inhibit *B.* bassiana infection of *D. antiqua* larvae (15), and K. oxytoca B313 (GenBank accession no. MF084958), which has no effect on the conidial germination and mycelial growth of *B.* bassiana BB1101 nor on the infection of *D. antiqua* larvae by *B.* bassiana according to a preliminary experiment (Fig. S6), were originally isolated from field-collected *D. antiqua* larvae via a culture-dependent method and preserved in 25% glycerol in our laboratory at −80°C. Standard chemicals and reagents including hypoxanthine (CAS no. 68-94-0), glutaric acid (CAS no. 110-94-1), kynurenic acid (CAS no. 492-27-3), phenyllactic acid (CAS no. 828-01-3), thymine (CAS no. 65-71-4), ethylmalonic acid (CAS no. 601-75-2), adipic acid (CAS no. 124-04-9), indoleacetic acid (CAS no. 87-51-4), picolinic acid (CAS no. 98-98-6) and ketoisocaproic acid (CAS no. 816-66-0) were purchased from Merck KGaA, Darmstadt, Germany.

10.1128/mSystems.00778-20.7 FIG S6 Conidial germination (a) and mycelial growth (b) of *B.* bassiana BB1101 and infection of *D. antiqua* larvae by *B.* bassiana BB1101 (c) under the effect of K. oxytoca B313. Values for each bar plot represent the conidial germination and mycelial growth rate relative to the control (± SD). Different letters above each bar refer to significant differences of multiple comparisons by the Tukey method within each set of bars (*n* = 6 for each treatment, “Control” = LB medium in panel a, and “Control” = 0 in panel b). The larval survival was estimated with Kaplan-Meier analysis (*n* = 40 for each group of larvae, one representative experiment of three, log rank test, α = 0.05), and “*” denotes a significant difference between the two connected lines in c (*P* < 0.05). “Axenic” refers to the larvae without treatments. “Axenic+Bb” refers to the larvae treated with *B.* bassiana BB1101 conidial suspension. “Axenic+B313” refers to the larvae treated with the bacterial suspension of K. oxytoca B313. “Axenic+Bb+B313” refers to the larvae treated with both the bacterial suspension of K. oxytoca B313 and the conidial suspension of *B.* bassiana BB1101. Download FIG S6, TIF file, 0.3 MB. Copyright © 2020 Zhou et al. 2020 Zhou et al. This content is distributed under the terms of the Creative Commons Attribution 4.0 International license.

10.1128/mSystems.00778-20.9 TABLE S1 Artificial diets for axenic *D. antiqua* larvae. Download Table S1, DOCX file, 0.02 MB. Copyright © 2020 Zhou et al. 2020 Zhou et al. This content is distributed under the terms of the Creative Commons Attribution 4.0 International license.

### Experiment I: effects of selected bacterial strains on *B.* bassiana infection of *D. antiqua* larvae, conidial germination, and mycelial growth of *B.* bassiana BB1101.

The effects of the six bacterial strains and K. oxytoca B313 on the survival of axenic larvae were determined. For each bacterial strain, 4 ml of overnight lysogeny broth (LB) cultures (28°C and rotary shaking at 150 rpm for 12 h) was centrifuged at 3,000 × *g* for 5 min to collect bacterial cells, and then the bacterial cells were washed three times with phosphate-buffered saline (PBS) and resuspended in PBS (10^6^ CFU/ml). Subsequently, 200 μl of the suspension was added to sterilized filter paper in a 30-mm petri dish, and three surface-sterilized *D. antiqua* eggs were placed on the paper. After hatching, artificial diets (containing no antibiotics) were put inside the petri dish to feed the hatched larvae at 24°C in darkness. For the control group, 200 μl of PBS was used in place of the bacterial cell suspension. Subsequently, one hatched larva from each petri dish was picked out and reared with artificial diets containing no antibiotics for further observation. Ninety petri dishes were set up for each bacterial strain (45 for bacterial suspension and 45 for PBS). Those larvae were incubated at 24°C in darkness, and larval survival was recorded every 2 days until pupation.

The effects of the six bacterial strains and K. oxytoca B313 on entomopathogenic infection of *D. antiqua* larvae with *B.* bassiana BB1101 were compared. For the comparison between each bacterial strain and K. oxytoca B313, overnight LB cultures (28°C and rotary shaking at 150 rpm for 12 h) for each mentioned bacterial strain or K. oxytoca B313 were centrifuged at 3,000 × *g* for 5 min, and then the bacterial cells were collected, washed, and resuspended in PBS (10^6^ CFU/ml). Moreover, a conidial suspension of *B.* bassiana BB1101 was obtained by washing the fungal plate with sterilized water containing 0.05% Tween 80 and filtered through a sterilized multilayer gauze, and the suspension was adjusted to 10^6^ CFU/ml with sterilized water containing 0.05% Tween 80. A volume of 200 μl bacterial suspension of each bacterial strain or B313 was added into a 30-mm petri dish containing three surface-sterilized *D. antiqua* eggs placed on sterilized filter paper, and the hatched larvae were reared with artificial diets (containing no antibiotics) for 1 week (second instar).

Subsequently, one of the three larvae was randomly selected, taken out, and sprayed with the above conidial suspension and air dried on a piece of sterilized filter paper. Then, the larva was reared with an artificial diet containing no antibiotics for further observation (Bb+bacterial strain). Forty-five larvae (one hatched larva from each petri dish containing three larvae) treated with one of the six bacterial strains or K. oxytoca B313 were individually collected and sprayed with *B.* bassiana BB1101 conidial suspension. In addition, another group of axenic larvae (45 larvae) were treated with the same conidial suspension one by one without inoculation of bacteria (Bb). Then, for each comparison, each larva from these three groups (Bb, selected bacterial strain+Bb, and B313+Bb) was separately fed with artificial diets (containing no antibiotics). Those larvae were incubated at 24°C in darkness, and larval survival was recorded every 2 days until pupation as described above. A preliminary test showed that *B.* bassiana BB1101 has excellent insecticide activity against axenic larvae (Fig. S6), so the comparison between axenic larvae treated by *B.* bassiana BB1101 conidial suspension and those without treatment was not set up in each comparison.

The effect of the six bacterial strains and K. oxytoca B313 on the conidial germination and mycelial growth of *B.* bassiana BB1101 was determined using a previously described method with minor modifications (38). A conidial suspension of *B.* bassiana BB1101 (10^6^ CFU/ml) was obtained as described in the previous study. LB cultures of each bacterial strain (incubated for 72 h at 28°C with rotary shaking at 150 rpm) were centrifuged at 3,000 × *g* for 5 min, and the supernatant was filtered with a 0.20-μm filter {polytetrafluoroethylene (PTFE) syringe filter [catalog no. SCAA-1114; ANPEL Laboratory Technologies (Shanghai) Inc.]} and diluted by 1×, 5×, and 25× using LB medium. For each strain, 100 μl of the conidial suspension was combined with 3.9 ml of diluted LB culture supernatant and then incubated at 25°C with rotary shaking at 180 rpm for 24 h in darkness. LB medium instead of the diluted supernatant was used in the control group. Conidia were defined as germinated when the length of the germ tube was greater than or equal to the conidia under a microscope (39). Each test for one specific bacterial strain on conidial germination was repeated six times. The conidial germination rate was presented as the percent value relative to that of the control.

To determine the effects of each individual strain on the mycelial growth of *B.* bassiana BB1101, each bacterial strain was cultured in LB medium for 12 h at 28°C with rotary shaking at 150 rpm, and then the culture was centrifuged at 3,000 × *g* for 5 min to collect bacterial cells. Subsequently, these cells were washed three times with PBS and resuspended to 100, 20, and 4 CFU/ml. Five milliliters of the bacterial cell suspension at different concentrations was added to 15 ml of melted potato dextrose agar (PDA; stored at 50°C in a water bath after autoclaving) and then mixed and poured into a 90-mm petri dish (approximately 500, 100, and 20 CFU/petri dish). These plates were used in the treatment group. For the control group, 5 ml of PBS was used in place of the bacterial cell suspensions. Agar plugs (3 mm, without conidia) taken from the leading edge of the *B.* bassiana BB1101 culture growing on 1/4 PDA plates were inoculated in the center of the PDA plates containing bacterial cells (the treatment groups) or without bacterial cells (the control group), and agar plugs from the same plate were randomly assigned to different treatment groups. Each petri dish was regarded as one replicate, and each test for one specific bacterial strain on mycelial growth was repeated six times. The plates were subsequently incubated at 25°C for 10 days in darkness. Starting the next day, the diameter of fungal mycelia was measured each day in two directions at right angles to each other during the experimental period. The mycelial growth rate was calculated as the percent value relative to that of the control.

### Experiment II: metabolomic analysis of the six bacterial strains and K. oxytoca B313 metabolites.

Individual bacterial LB cultures (rotary shaking at 150 rpm and 28°C for 72 h) for each strain including K. oxytoca B313 were centrifuged at 3,000 × *g* for 5 min. Subsequently, 20 μl of supernatant filtered with a 0.22-μm PTFE syringe filter [catalog no. SCAA-1114; ANPEL Laboratory Technologies (Shanghai) Inc.] was mixed with 120 μl of precooled 50% methanol (4°C for 24 h), vortexed for 1 min, and incubated at room temperature for 10 min. The mixture was then stored overnight at −20°C and centrifuged at 4,000 × *g* for 20 min, and the supernatants were transferred separately into new glass vials for the liquid chromatography-mass spectrometry (LC-MS) analysis. LB medium was also extracted as described above and analyzed with LC-MS to subtract the background noise of LB. In addition, pooled quality control (QC) samples were also prepared by combining 10 μl of each extraction sample. Six replicates were used for each group (B585, B263, B108, B505, B253, B424, B313, LB, and QC).

Each sample from all nine groups was analyzed using an LC-MS system according to a previously published method with minor modifications (40). An Acquity ultraperformance liquid chromatography (UPLC) ethylene-bridged hybrid (BEH) amide column (100 mm × 2.1 mm, 1.7 μm; Waters, United Kingdom) was used, with a mobile phase of solvent A (25 mM ammonium acetate) and solvent B (isopropanol:acetonitrile = 9:1 + 0.1% formic acid). Gradient elution conditions were as follows: 0 to 0.5 min, 95% B; 0.5 to 9.5 min, 95% to 65% B; 9.5 to 10.5 min, 65% to 40% B; 10.5 to 12 min, 40% B; 12 to 12.2 min, 40% to 95% B; and 12.2 to 15 min, 95% B. The instrumentation conditions of a high-resolution tandem mass spectrometer, TripleTOF 5600 Plus (Sciex, United Kingdom), were set up according to previously published methods (41). Metabolomic data were analyzed using the XCMS software package (https://sciex.com/products/software/xcms-plus-software) according to methods published previously (42). Metabolites were annotated if the mass difference between the observed metabolite and the one from databases (Kyoto Encyclopedia of Genes and Genomes Database and the Human Metabolome Database) was less than 10 ppm. Besides, an in-house fragment spectrum library of metabolites was used to identify the metabolite. Principal-component analysis (PCA) was performed for outlier detection and batch effects evaluation with the preprocessed data set. Supervised partial least-squares discriminant analysis (PLS-DA) was conducted to discriminate the different variables between groups with calculated variable importance of projection (VIP) values. A VIP cutoff value of 2 was used to select important features.

To subtract the background noise from LB medium, additional data analysis procedures were conducted (Fig. S7). A data set union was created by merging metabolites from each bacterial sample group which were significantly upregulated at least 2-fold compared with the LB medium (step I in Fig. S7). Subsequently, a more critical parameter (metabolites produced by the selected bacterial strains upregulated at least 10 times compared with the B313 group) was used to ensure that the obtained metabolites were produced by the six strains and of high antifungal activity (step II in Fig. S7). Metabolomics data were deposited in the EMBL-EBI MetaboLights database (43).

10.1128/mSystems.00778-20.8 FIG S7 Additional data analysis procedures to subtract the background noise from LB medium. A data set union was created by merging metabolites from each bacterial sample group that were significantly upregulated by at least 2 times compared with LB medium (step I). Subsequently, a more critical parameter (metabolites produced by the selected bacterial strains upregulated at least 10 times compared with the B313 group) was used to ensure that the obtained metabolites were produced by the six strains and had high antifungal activity (step II). Download FIG S7, TIF file, 2.3 MB. Copyright © 2020 Zhou et al. 2020 Zhou et al. This content is distributed under the terms of the Creative Commons Attribution 4.0 International license.

### Experiment III: quantification of candidate metabolites on the body surface of axenic and nonaxenic *D. antiqua* larvae.

The difference between laboratory standard conditions and *in situ* field environments with bacteria may lead to variation in concentrations and compositions of bioactive metabolites that protect *D. antiqua* larvae from *B.* bassiana infection. Moreover, metabolomic analysis cannot provide *in situ* information, particularly concentrations of candidate metabolites. Thus, a quantification experiment of candidate metabolites, including ketoisocaproic acid, glutaric acid, adipic acid, phenyllactic acid, indoleacetic acid, kynurenic acid, picolinic acid, ethylmalonic acid, hypoxanthine, and thymine, on the body surface of *D. antiqua* second-instar larvae was conducted. In addition, the concentrations of the candidate metabolites on the body surface of axenic and nonaxenic larvae were compared.

To quantify the candidate metabolites on the body surface of nonaxenic *D. antiqua* larvae, 20 field-collected second-instar larvae from five randomly selected garlic plants (four larvae from each plant) were collected, and the frass on the body surface of the larvae was cleaned with a little brush. Then all 20 larvae were put into one centrifuge tube (2 ml), mixed with 1.5 ml of double-distilled water (ddH_2_O), and sonicated for 30 s at 50°C. Subsequently, the sample was vortexed for 60 s, and the liquid inside the tube was collected and centrifuged at 3,000 × *g* for 10 min in another centrifuge tube. The supernatant was collected and filtered with a 0.22-μm PTFE syringe filter [catalog no. SCAA-1114; ANPEL Laboratory Technologies (Shanghai) Inc.], followed by quantification of the candidate metabolites. Detailed information on instrumentation and detection conditions is in Text S1 in the supplemental material. Another group of 20 axenic larvae at the second instar (surface-sterilized eggs reared with artificial diets containing no antibiotics for 7 days) was also collected. The body surface samples for quantification of candidate metabolites of axenic *D. antiqua* larvae were prepared as described above. According to the quantity of the selected metabolites in the supernatant from 20 *D. antiqua* larvae, the quantity of selected metabolites for each larva was calculated. Previous preliminary experiments showed that there was about 0.0920 ± 0.047 mg H_2_O on the body surface of second-instar *D. antiqua* larva (unpublished data; average ± standard deviation). Thus, the actual concentration for each selected metabolite on the body surface of *D. antiqua* larva was estimated. Each group of 20 larvae was regarded as one replicate, and the above two tests were repeated five times.

10.1128/mSystems.00778-20.1 TEXT S1 Supplemental methods for quantification of candidate metabolites on the body surface of axenic and nonaxenic *D. antiqua* larvae. Download Text S1, PDF file, 0.2 MB. Copyright © 2020 Zhou et al. 2020 Zhou et al. This content is distributed under the terms of the Creative Commons Attribution 4.0 International license.

### Experiment IV: effects of bacterial metabolite cocktail on conidial germination and mycelial growth of *B.* bassiana BB1101.

In experiment III, the concentrations of selected metabolites (ketoisocaproic acid, glutaric acid, adipic acid, phenyllactic acid, indoleacetic acid, kynurenic acid, picolinic acid, ethylmalonic acid, hypoxanthine, and thymine) on the larval body surface of both axenic and nonaxenic larvae were determined. Based on these results, two metabolite cocktails for body surface of axenic and nonaxenic larvae were prepared separately to perform *in situ* simulation. For each of the above cocktails, a serial concentration of 2^1^, 2^0^, 2^−1^, 2^−2^, 2^−3^, 2^−4^, 2^−5^, and 2^−6^ times the actual concentration for each metabolite was set. Details of the dosage setup of selected metabolites are in Table S2. Effects of the above bacterial metabolite cocktails on conidial germination and mycelial growth of *B.* bassiana BB1101 were determined as follows.

10.1128/mSystems.00778-20.10 TABLE S2 Detailed dosage setup of selected metabolites for metabolite cocktails of body surface of axenic and nonaxenic larvae. Download Table S2, DOCX file, 0.02 MB. Copyright © 2020 Zhou et al. 2020 Zhou et al. This content is distributed under the terms of the Creative Commons Attribution 4.0 International license.

To determine the effects of bacterial metabolite cocktails on conidial germination of *B.* bassiana BB1101, a series of concentrations of solutions (Table S2) for each cocktail was prepared with sterilized 1/4 potato dextrose broth (PDB) and filtered with 0.22-μm PTFE syringe filters [catalog no. SCAA-1114; ANPEL Laboratory Technologies (Shanghai) Inc.]. According to previous reports (44, 45), 1/4 PDB or PDA was used. Subsequently, 10 μl of *B.* bassiana BB1101 conidial suspension (10^6^ CFU/ml) obtained as described above was added into a test tube containing 4 ml of the above PDB-metabolite cocktail. Test tubes containing the above mixture were incubated at 25°C with rotary shaking at 180 rpm for 24 h in darkness. Conidial germination was determined via microscopy as described for experiment I. For each metabolite cocktail, the test for each dose was repeated six times, and 1/4 PDB medium without metabolites was used as a control. The conidial germination rate was presented as the percent value relative to that of the control.

To test the effect of bacterial metabolite cocktails on mycelial growth of *B.* bassiana BB1101, an aliquot of individual metabolite cocktail stock solution {filtered using a 0.22-μm PTFE syringe filter [catalog no. SCAA-1114; ANPEL Laboratory Technologies (Shanghai) Inc.]} was added into 10 ml 1/4 soft PDB agar inside a petri dish to final concentrations as described in Table S2. Agar plugs (3 mm) taken from the leading edge of the *B.* bassiana BB1101 culture growing on 1/4 PDA plates (without conidia) were inoculated into the center of the PDA plates that contained different doses of metabolite cocktails (for the treatment groups). For the control group, 1/4 PDB was used instead of the metabolite cocktail solution. For each metabolite cocktail, the test for each dose was repeated six times, and agar plugs from the same *B.* bassiana BB1101 culture PDA plate were randomly assigned to different treatment groups. Subsequently, these plates were incubated at 25°C in darkness for 10 days. The diameter of fungal mycelia was measured every 2 days in two directions at right angles to each other during the experiment. The mycelial growth rate was calculated as the percent value relative to that of the control group.

### Experiment V: effects of candidate metabolite cocktail on fungal infection of *D. antiqua* larvae with *B.* bassiana BB1101.

A bacterial metabolite cocktail containing ketoisocaproic acid (66.1 mg/liter), glutaric acid (63.7 mg/liter), adipic acid (587.5 mg/liter), phenyllactic acid (6.3 mg/liter), indoleacetic acid (191.2 mg/liter), kynurenic acid (0.1 mg/liter), and picolinic acid (0.2 mg/liter) was prepared according to the concentration of each metabolite from the nonaxenic larval body surface. One artificial diet containing the above organic mixture was prepared by adding organic acid solutions to sterilized diets at 55°C; antibiotics were not added into the diets. Final concentrations for each organic acid in the diet were the same as that in the above metabolite cocktail. To detect the effect of the cocktail on *B.* bassiana infection of axenic *D. antiqua* larvae, 2 ml of the cocktail was added to sterilized filter paper in a 90-mm petri dish. An axenic second-instar *D. antiqua* larva was sprayed with the above cocktail on sterilized filter paper and air dried, and the larva was sprayed with a *B.* bassiana BB1101 conidial suspension (10^6^ conidia/ml, cocktail+Bb) as described above. Then the larva was put inside the petri dish containing a piece of cocktail-soaked filter paper and fed with the artificial diets containing the seven organic acids.

To detect the effect of the cocktail on survival of axenic *D. antiqua* larvae, one second-instar axenic larva was treated with the cocktail combined with sterilized water instead of the *B.* bassiana BB1101 conidial suspension, put inside a petri dish containing a piece of cocktail-soaked filter paper, and fed with artificial diets containing the seven organic acids (cocktail). To detect the effect of *B.* bassiana on survival of axenic *D. antiqua* larvae, one second-instar axenic larva was treated with sterilized water instead of the cocktail, further treated with the *B.* bassiana BB1101 conidial suspension, put inside a petri dish containing a piece of water-soaked filter paper, and fed with artificial diets containing neither organic acids nor antibiotics (Bb). For the control (Blank), one axenic larva without treatment was incubated inside a petri dish containing water-soaked filter paper and fed with artificial diets containing neither organic acids nor antibiotics. All larvae were incubated in darkness at 24°C. Larval survival was recorded every 2 days until pupation.

### Data analysis.

Data analysis was conducted with IBM SPSS 20.0 (International Business Machines Corp., Armonk, NY, USA). The normality and homogeneity of observed variances were tested with the Kolmogorov-Smirnov test and Levene’s test, respectively. Larval survival rates under various treatments were compared with Kaplan-Meier analysis (log rank test) in experiments I and IV. Conidial germination or mycelial growth data in experiments I and IV were compared with one-way ANOVA followed by Tukey’s multiple comparisons, Welch’s ANOVA followed by Dunnett’s T3 test, or the Kruskal-Wallis test depending on the normality and homogeneity of the variances. In experiment III, quantifications of candidate metabolites on the body surface of axenic and nonaxenic larvae were compared using the independent *t* test or Mann-Whitney U test. The figures for the above-described experiments were produced using SigmaPlot 12.5 (Systat Software Inc., San Jose, CA, USA).

### Data availability.

All data during the study are available from the corresponding author by request. Metabolomics data have been deposited in the EMBL-EBI MetaboLights database (https://doi.org/10.1093/nar/gkz1019; PMID: 31691833) with the identifier MTBLS2074.
