# Supplementary figures and images for: Symbiotic Bacterium-Derived Organic Acids Protect Delia antiqua Larvae from Entomopathogenic Fungal Infection
Source: mSystems. 2020 Nov 17;5(6):e00778-20. doi: 10.1128/mSystems.00778-20 (PMC7677000; doi:10.1128/mSystems.00778-20)

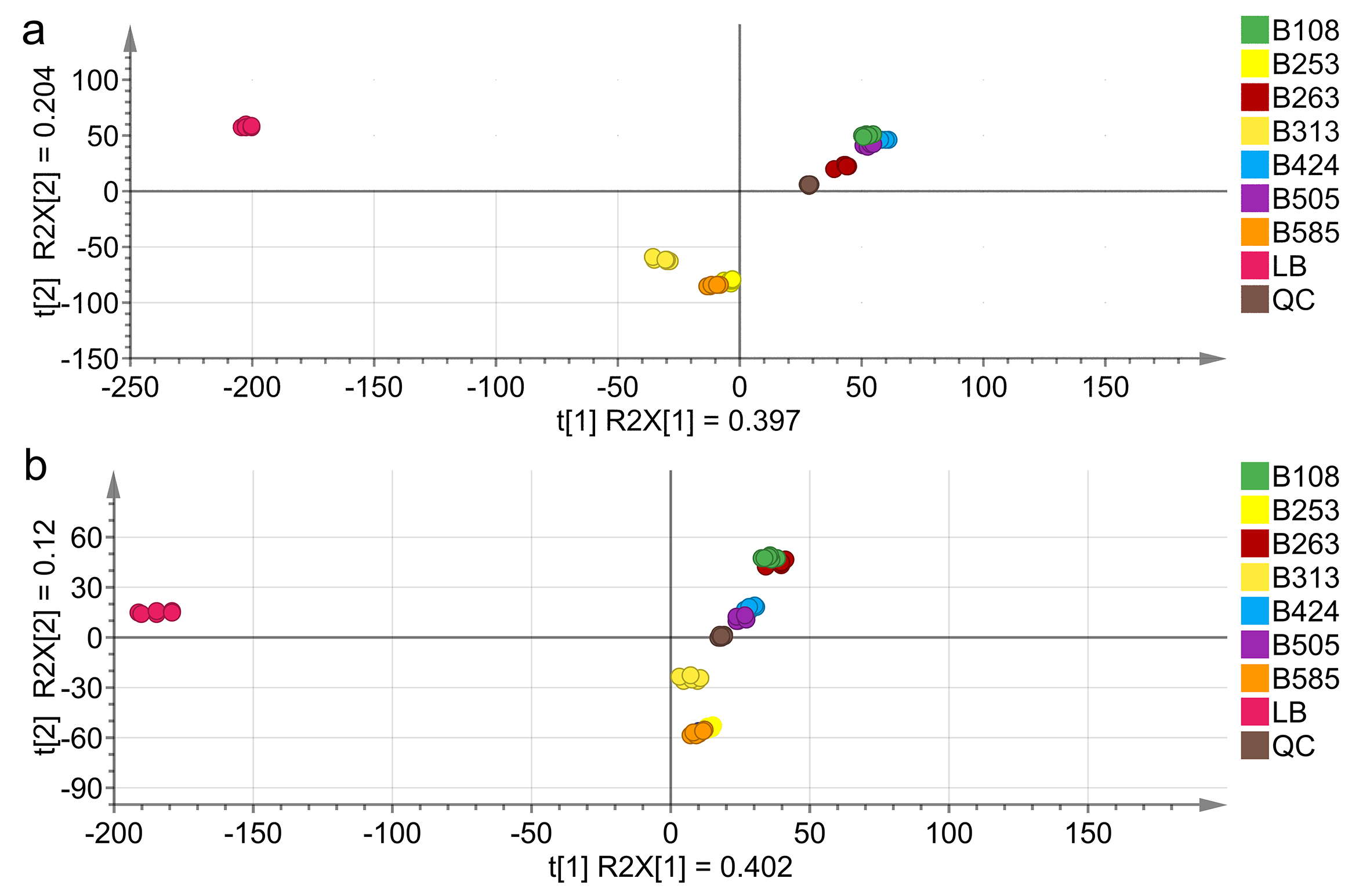

Supplement: FIG S3 [file mSystems.00778-20-sf003.tif]

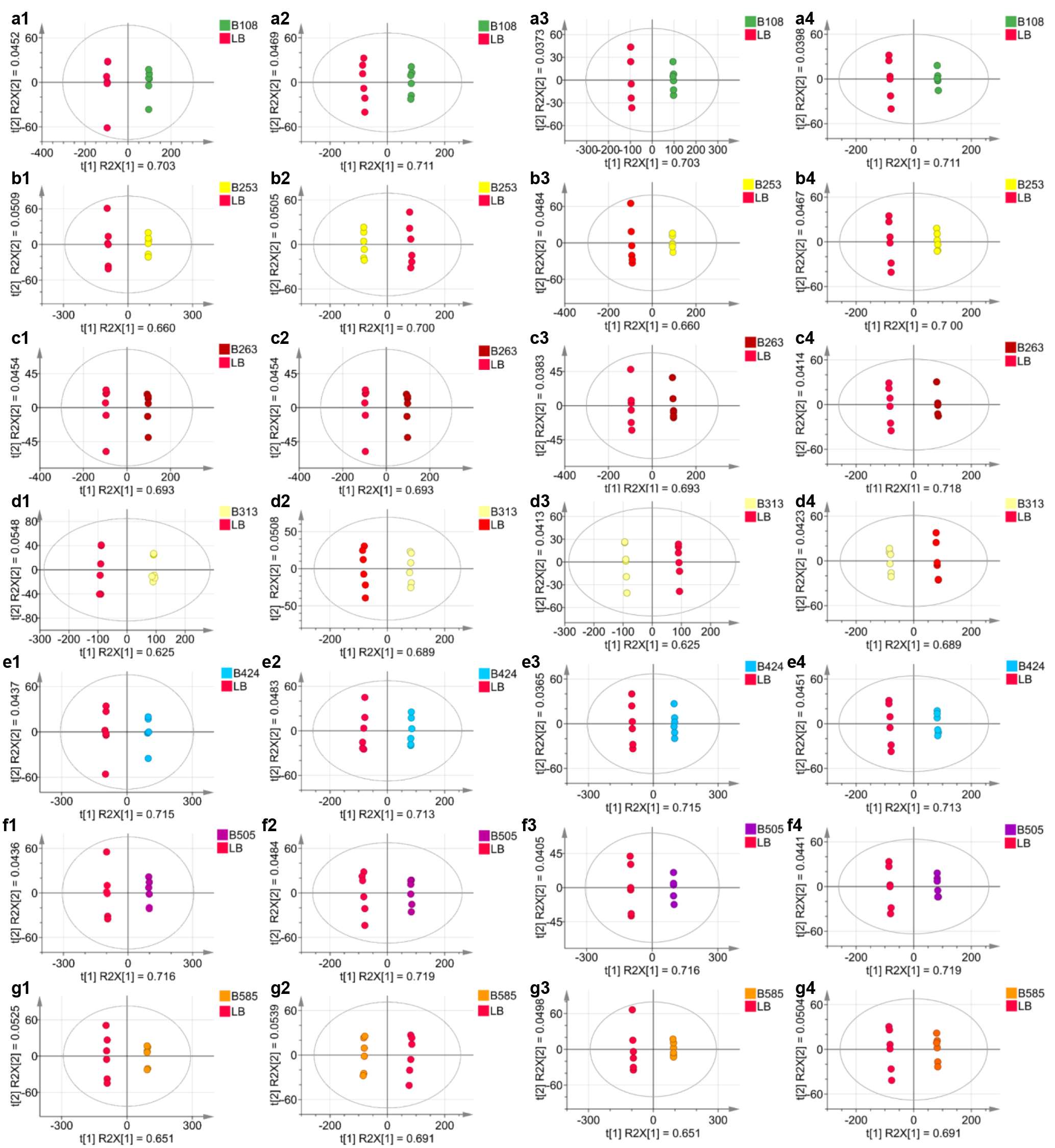

Supplement: FIG S4 [file mSystems.00778-20-sf004.tif]

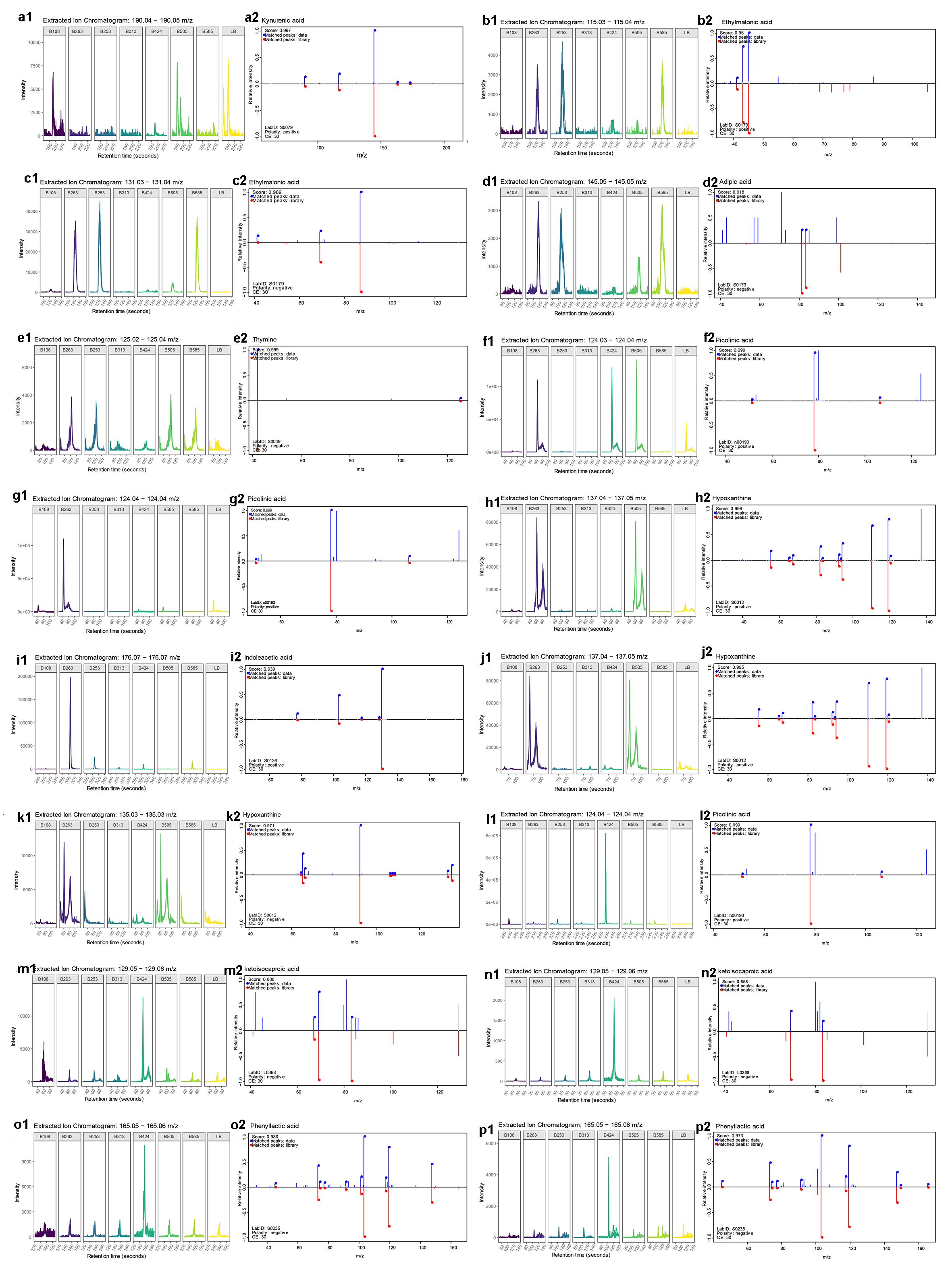

Supplement: FIG S5 [file mSystems.00778-20-sf005.tif]
