# Supplementary material for: Symbiotic Bacterium-Derived Organic Acids Protect Delia antiqua Larvae from Entomopathogenic Fungal Infection
Source: mSystems. 2020 Nov 17;5(6):e00778-20. doi: 10.1128/mSystems.00778-20 (PMC7677000; doi:10.1128/mSystems.00778-20)
Supplement: TABLE S1 [file mSystems.00778-20-st001.docx]

**Table S1** Artificial diets for axenic *D. antiqua* larvae^*^

| Ingredient | Dosage (g/750 mL H_2_O) | Producer |
| --- | --- | --- |
| Rat feed | 48 | Biotech-hd Biotechnology Co. LTD |
| Defatted soybean powder | 56 | Solarbio Life Sciences |
| Cellulose powder | 40 | Herbalife International of America, Inc. |
| Sucrose | 16 | Solarbio Life Sciences |
| Agar | 16 | Solarbio Life Sciences |
| Yeast extract | 16 | Solarbio Life Sciences |
| Methylparaben | 3 | Merck KGaA, Darmstadt, Germany |
| Chloramphenicol | 1.5 | Merck KGaA, Darmstadt, Germany |
| Penicillin G sodium salt | 1,5 | Solarbio Life Sciences |
| Neomycin sulphate | 1.5 | Merck KGaA, Darmstadt, Germany |
| Choline chloride | 1.2 | Merck KGaA, Darmstadt, Germany |
| Vitamin C | 1.2 | Solarbio Life Sciences |

* Water, rat feed, defatted soybean powder, cellulose powder, sucrose, agar, and yeast extract were mixed in water and sterilized at 121 °C for 20 min, and methylparaben, chloramphenicol (dissolved in ethanol), penicillin G sodium salt, neomycin sulphate, choline chloride and vitamin C were added into the above mixture after sterilization.
