# Supplementary material for: Symbiotic Bacterium-Derived Organic Acids Protect Delia antiqua Larvae from Entomopathogenic Fungal Infection
Source: mSystems. 2020 Nov 17;5(6):e00778-20. doi: 10.1128/mSystems.00778-20 (PMC7677000; doi:10.1128/mSystems.00778-20)
Supplement: TEXT S1 [file mSystems.00778-20-s0001.pdf]

Supplementary methods for "Symbiotic bacteria-derived organic acids protect *Delia antiqua* larvae from entomopathogenic fungal infection"

Quantification of water on the body surface of 2nd instar *D. antiqua* larvae

To quantify the water on the body surface of *D. antiqua* larvae, six groups of 2nd instar larvae (reared for seven days with garlic and almost the same size, ten for each group) were collected, and the frass attached to the larval body surface was removed with a little brush. Subsequently, each group of the above larvae were weighed inside a sealed 2.0 mL Eppendorf tube, and the net weight of the group of larvae was calculated (initial weight). After that, the group of larvae were put inside a 90 mm Petri dish and air dried. Then, these larvae were weighed every 5 min until the weight remained stable (final weight). For each group of ten larvae, the weight of water on larval body surface for each larva was calculated as follow:

$$\text{Water on larval body surface for each larva} = (\text{final weight} - \text{initial weight})/10$$

An average of the water weight on the larval body surface for each larva from the six groups was calculated.

Quantification of ketoisocaproic acid, glutaric acid, adipic acid, phenyllactic acid, indoleacetic acid, kynurenic acid, picolinic acid, and ethylmalonic acid

Sample preparation

Samples for quantification of selected organic acids from the larval body surface were prepared as follows before quantitive detection. One-hundred microliters of the samples were combined with 400 μL of protein precipitator solution and vortexed for 1 min.

Subsequently, the mixture was centrifuged at 13,200 ×g for 4 min. The supernatant was collected for further quantitive detection. Moreover, a series of standard water solutions for ketoisocaproic acid, glutaric acid, adipic acid, phenyllactic acid, indoleacetic acid, kynurenic acid, picolinic acid, and ethylmalonic acid at doses of 20, 100, 500, 2,500, 12,500, and 25,000 μg/L were prepared for further quantification in actual samples.

Instrumentation and detection conditions

The test was conducted with a HPLC-MS/MS system including the LC chromatography system (Dionex's UltiMate 3000) and a mass spectrometer (AB MDS SCIEX 3200Q TRAP). A MSLab50AA-C18 (150 mm×4.6 mm, 5 μm) column was used for LC chromatography separation. The column temperature was set at 50 °C, and the flow-rate of mobile phase conditions was 1 mL/min. Mobile phase including (A) ammonium acetate (1 mM) and (B) acetonitrile was filtered through a 0.22 μm poly tetra fluoroethylene (PTFE) syringe filter (Cat. No. SCAA-1114, ANPEL Laboratory Technologies (Shanghai) Inc.) and degassed ultrasonically before use. The solvent elution program was as follows: 0-1 min, 5% B; 1-1.1 min, 5-50% B; 1.1-12 min, 50-70% B; 12-12.1 min, 70-100% B; 12.1-15 min, 100% B; 15-15.1 min, 100-5% B; 15.1-20 min, 5% B. The injection volume was 5 μL. Mass spectrometric detection was operated using electrospray ionization (ESI) source in negative ion detection. Quantification was performed using multiple reactions monitoring (MRM). The optimized ionspray (IS) voltage was set at -4,500V. Source temperatures were maintained at 500 ºC. Nitrogen was used as nebulizing gas (55 psi), auxiliary gas (60

psi) and curtain gas (20 psi). Entrance potential (EP) was -10V. The collision cell exit potential was set as -2.0, and collision gas was set as medium.

Quantification of hypoxanthine and thymine

Sample preparation

Samples for quantification of hypoxanthine and thymine from the larval body surfaces were prepared as follows before quantitive detection. One-hundred microliters of the samples were combined with 400 μL of protein precipitator solution and vortexed for 1 min. Subsequently, the mixture was centrifuged at 13,200 × g for 4 min. The supernatant was collected and vacuum dried. The residue was dissolved in 400 μL methanol. A series of standard methanol solutions for hypoxanthine and thymine at doses of 4, 20, 100, 500, and 2,500 μg/L were prepared for further quantification in actual samples.

Instrumentation and detection conditions

The test was conducted with a LCMS-2010 (Shimadzu Kyoto, Japan). The column utilized for separation was a Shimadzu VP-ODS column (2.0 mm×150 mm, 5 μm). The analytical column was protected by a C18 guard-pak cartridge (Waters, Milford, MA, USA). Mobile phase including (A) ammonium acetate (40 mM) and (B) methanol was degassed ultrasonically before use. Each component of the mobile phase was filtered through a 0.22 μm PTFE syringe filter (Cat. No. SCAA-1114, ANPEL Laboratory Technologies (Shanghai) Inc.). Chromatographic separation was performed at 25 °C and at a flow-rate of 0.2 mL/min. The solvent elution program was as follows: 0-10 min, 5-15% B; 10-15 min, 15% B; 15-18 min, 15-20% B; 18-23 min, 20% B; 23-28

min, 20-5% B. The wavelength of the photodiode array detector was 190–300 nm. The amount of injection was 5 μL. MS coupling with an ESI interface was used. Peaks were detected in both scan and SIM mode. Mass spectrometric detection conditions for both scan and SIM are as follows. ESI temperature was 400 °C. Curved desolvation line (CDL) and block temperatures were 250 and 200 °C, respectively. Probe voltage was +4,500 V. Detector voltage was 1,500 V. CDL voltage was −20 V. Q-array Bios was 50 V. Nebulizing gas flow was 4.5 L/min.
