# Supplementary material for: Symbiotic Bacterium-Derived Organic Acids Protect Delia antiqua Larvae from Entomopathogenic Fungal Infection
Source: mSystems. 2020 Nov 17;5(6):e00778-20. doi: 10.1128/mSystems.00778-20 (PMC7677000; doi:10.1128/mSystems.00778-20)
Supplement: TABLE S2 [file mSystems.00778-20-st002.docx]

**Table S2** Detailed dosage setup of selected metabolites for metabolite cocktails of body surface of axenic and nonaxenic larvae

| larvae | Times relative to actual concentration | Chemical concentration (mg/L) | | | | | | |
| --- | --- | --- | --- | --- | --- | --- | --- | --- |
|  |  | Ketoisocaproic acid | Glutaric acid | Adipic acid | Phenyllactic acid | Indoleacetic acid | Kynurenic acid | Picolinic acid |
| nonaxenic | 2^1^ | 132.2 | 127.5 | 1175.0 | 12.6 | 382.4 | 0.2 | 0.4 |
|  | 2^0^ * | 66.1 | 63.7 | 587.5 | 6.3 | 191.2 | 0.1 | 0.2 |
|  | 2^-1^ | 33.1 | 31.9 | 293.7 | 3.2 | 95.6 | 0.1 | 0.1 |
|  | 2^-2^ | 16.5 | 15.9 | 146.9 | 1.6 | 47.8 | 0.0 | 0.0 |
|  | 2^-3^ | 8.3 | 8.0 | 73.4 | 0.8 | 23.9 | 0.0 | 0.0 |
|  | 2^-4^ | 4.1 | 4.0 | 36.7 | 0.4 | 12.0 | 0.0 | 0.0 |
|  | 2^-5^ | 2.1 | 2.0 | 18.4 | 0.2 | 6.0 | 0.0 | 0.0 |
|  | 2^-6^ | 1.0 | 1.0 | 9.2 | 0.1 | 3.0 | 0.0 | 0.0 |
|  | Control | 0.0 | 0.0 | 0.0 | 0.0 | 0.0 | 0.0 | 0.0 |
| axenic | 2^1^ | 9.7 | 17.5 | 4.9 | 28.0 | 23.8 | 1.1 | 0.4 |
|  | 2^0^ * | 4.9 | 8.7 | 2.4 | 14.0 | 11.9 | 0.5 | 0.2 |
|  | 2^-1^ | 2.4 | 4.4 | 1.2 | 7.0 | 6.0 | 0.3 | 0.1 |
|  | 2^-2^ | 1.2 | 2.2 | 0.6 | 3.5 | 3.0 | 0.1 | 0.1 |
|  | 2^-3^ | 0.6 | 1.1 | 0.3 | 1.8 | 1.5 | 0.1 | 0.0 |
|  | 2^-4^ | 0.3 | 0.5 | 0.2 | 0.9 | 0.7 | 0.0 | 0.0 |
|  | 2^-5^ | 0.2 | 0.3 | 0.1 | 0.4 | 0.4 | 0.0 | 0.0 |
|  | 2^-6^ | 0.1 | 0.1 | 0.0 | 0.2 | 0.2 | 0.0 | 0.0 |
|  | Control | 0.0 | 0.0 | 0.0 | 0.0 | 0.0 | 0.0 | 0.0 |

“*” in the table refer to actual concentrations of each metabolite detected in corresponding samples.
